# Atlantic salmon skin barrier functions gradually enhance after seawater transfer

**DOI:** 10.1038/s41598-018-27818-y

**Published:** 2018-06-22

**Authors:** Christian Karlsen, Elisabeth Ytteborg, Gerrit Timmerhaus, Vibeke Høst, Sigurd Handeland, Sven Martin Jørgensen, Aleksei Krasnov

**Affiliations:** 1 Nofima, Osloveien 1, 1430 Aas, Norway; 2 UNI Research, Nygårdsgaten 112, 5008 Bergen, Norway

## Abstract

Atlantic salmon farming operates with high production intensities where skin integrity is recognized as a central factor and indicator for animal health and welfare. In the described trial, the skin development and its immune status in healthy Atlantic salmon reared in two different systems, a traditional open net-pen system and a semi-closed containment system, were investigated. Freshwater smolts were compared to post-smolts after 1 and 4 months in seawater. Growth performance, when adjusted for temperature, was equal between the systems. Skin analyses, including epidermis and dermis, showed that thickness and mucus cell numbers increased in pace with the growth and time post seawater transfer (PST). Gene expression changes suggested similar processes with development of connective tissue, formation of extracellular matrix and augmented cutaneous secretion, changes in mucus protein composition and overall increased immune activity related to gradually enforced protection against pathogens. Results suggest a gradual morphological development in skin with a delayed recovery of immune functions PST. It is possible that Atlantic salmon could experience increased susceptibility to infectious agents and risk of diseases during the first post-smolt period.

## Introduction

Success and sustainable growth of the commercial Atlantic salmon (*Salmo salar*) aquaculture depend principally on fish health and welfare. Development of semi-closed containment systems (S-CCS) at sea is a promising strategy aiming at further expansion of the Atlantic salmon production in Norway^1^. In general, these systems are developed for the rearing of post-smolts during a limited period after seawater transfer. S-CCS technologies are targeted to better control the farm environment and improved protection against infectious diseases and parasites. Many of the S-CCS systems are designed with a deeper water-inlet (>20 m below the surface). Such tempered water may be beneficial during warmer summer periods and cold winters, but may also reduce growth and other metabolic processes the rest of the year. However, the deep water intake aim to prevent parasitic infections, mainly sea lice. It is currently unknown if these changes in production parameters, may bring an altered diversity, prevalence or load of known or new microparasites compared to the open systems. For example, skin ulcerations of Atlantic salmon post-smolts is a reoccurring problem^2,3^, and one of the problems anticipated to increase in S-CCS systems. Bacteria such as the psychrophilic *Moritella viscosa*, causing winter-ulcer disease in farmed salmonids^4^, may increase the risk of skin ulcerations. Assessment of S-SSC, their advantages and drawbacks in comparison with traditional facilities requires systematic monitoring and multidisciplinary research.

The skin of fish is a protective and active layer of tissue that interacts with the surrounding environment^5^. It provides protection against external agents and has a high capacity for healing and regeneration^6^. Several cell types are involved in formation and maintenance of the cutaneous layer that forms the first physical and immune active barrier in fish^7–9^. The epidermis is a superficial multilayer of cells that forms a stratified epithelium. Mucus cells, scattered in the epidermis, secrete products with antimicrobial activities^10^. The keratocytes are the outermost cells, forming the epidermis cell layer which is in contact with the fish’s surroundings. Keratocytes rapidly migrate to cover wound surfaces with the ability to internalize particular matter as part of the nonspecific immune response^11,12^. Removal of this outer layer of fish skin may increase susceptibility to infections^7,9^. Underneath the epidermis is the dermis, which contains the fibrous connective tissue enriched with blood vessels. The outer part (*stratum spongiosum*) contains the scales. The deeper part (*stratum compactum*) has a high proportion of collagen fibers that provide the flexible support followed by vascularized tissue that links with the adipose tissue and the muscle. Skin structure under normal conditions and its ability to repair after damages are determined by diverse cellular elements, extracellular matrix (ECM) and function of mucus.

Atlantic salmon is poikilothermic, and biological processes will be affected by environmental parameters, such as water temperature. Temperature affects both specific and nonspecific immunity^13^. Low temperature may have systemic immunosuppressive effects on fish^14^ where protection from vaccination may decrease^15^. Effects on the nonspecific immunity may also be of great importance. Low temperatures decrease the motility of the re-epithelializing keratocytes^8^, while it may also enhance other responses such as e.g. lytic activities^16^, and potentially affect the many roles of mucus^10^. It is reported that skin of Atlantic salmon responded with enrichment of genes related to mucus immunity at low temperature^17^. A recent transcriptome study revealed systemic suppression of immunity in salmon during smoltification, which maintained at the same level after three weeks in seawater^18^. Further studies found substantial difference between salmon strains by the character and magnitude of immune changes during smoltification^19^. This might be associated with the increased occurrence of infectious diseases in salmon after seawater transfer^2,20^ as it remains unknown if expression of immune genes increases after initial down-regulation and how much time is required for recovery.

The goal of this study was assessment of conditions and temporal changes in salmon skin from smolt in freshwater to post-smolt in the first period after seawater transfer, and comparison between a S-CCS and conventional open sea net-pens. Performance of salmon from the same batch of smolts was tested in two different farming systems; in Preline S-CCS (Preline Fishfarming System AS, Norway) and in conventional open sea net-pens. While the water in Preline is heavily controlled by a deep water intake, the fish in open net-pens are constantly exposed to the natural water qualities at the site’s location. Skin was selected for its complex and dynamic barrier functions essential for integrity and protection of the Atlantic salmon. We performed histological examination and transcriptome analyses with focus on resistance, immunity and development of the skin. According to our results, it seems to be a generic response in salmon during the first period after seawater transfer, where immune functions are reduced. These results further indicate that it may be beneficial for salmon to spend the first months in a more controlled and confined environment.

## Material and Methods

### Fish and production systems

The field study was performed as a collaboration between Lerøy Seafood Group ASA and the Centre for Research-based Innovations in Controlled-environment Aquaculture, CtrlAQUA. Atlantic salmon (Salmobreed QTL duo) hatched in 2015 were reared at Sjøtroll Havbruk AS (Kjærelva, Fitjar, Norway). Smolts were produced by decreasing the day length from LD24:0 to LD12:12 for 8 weeks, followed by 8 weeks on LD24:0 before transfer by well boat to seawater as 1+ smolts in May 2016. The fish group was split and randomly placed into two different culturing systems. The S-CCS (with 157,126 fish) examined in this study is an in-sea 50 m floating semi-closed raceway system (Preline, Preline Fishfarming System AS, Norway) designed to cultivate Atlantic salmon during the first seawater period. The Preline S-CCS holds approximately 2000 m^3^ of water and was located in a region with a depth of 100 m. The intake-water is pumped from 30 m and propellers create a water current of 10–20 cm s-1 exchanging the water in the system within 5–6 min. This is in contrast to the traditional 160 m circular open water net-pen system (with 164,286 fish), located at a depth of 250 m, and with a 60 m deep pen which is exposed to the natural fluctuations in water current at the site’s location. Both sites were located in the West of Norway (S-CCS: latitude: 60° 20′ 527′′N, longitude: 5° 38′ 293′′E, and open net-pen: latitude: 59° 57′ 498′′N, 5° 49′ 238′′E). Oxygen, temperature and salinity were automatically monitored (OxyGuard Commander, Sterner, Bergen) with daily registration (Fishtalk, AkvaGroup, Bryne) at 3 m, 8 m and 15 m in the open net-pen system, and in the inlet and outlet water in the S-CCS. Environmental parameters during the experiment period at sea (May-August 2016) are presented in Supplementary Fig. S1. The fish in both systems were automatically fed by commercial freshwater/seawater dry diets (EWOS, Norway) and grew within normal commercial expectations. All husbandry practices at the farms were conducted in accordance with national guidelines regarding animal welfare in addition to standard protocols for Lerøy Vest AS. Fish health and welfare status was regularly monitored by independent fish health veterinarians during production.

### Sampling and growth parameters

Fish (n = 30, weight 101 ± 4.2 g and condition factor (CF) 1.15 ± 0.01 (mean ± SEM), Table 1) were sampled in freshwater two weeks before transfer to seawater systems. First sampling in seawater was performed 1 month post seawater transfer (PST) with consecutive days between the systems. The measured temperature during sampling was 9.8 °C (average) in open net-pen and 8.2 °C in S-CCS. The farms were re-sampled 4 months PST. Temperatures were 15.1 °C and 12.3 °C in net-pen and S-CCS, respectively. Fifteen fish were utilized per sample time-point (weight and CF in Table 1). Netted Atlantic salmon were killed by a lethal dose of NaCO_3_-buffered tricaine methanesulphonate (MS222, Sigma-Aldrich) anaesthetics and immediately sampled on site. All fish were individually weighed (to nearest g) and their length measured (to nearest 0.5 cm). Two pieces of skin were excised from the left side of each fish in the area posterior of the dorsal fin and above the lateral line. Sample for skin histology was added into 20 ml pots containing 10% buffered formalin (CellStor™ pots, CellPath) and stored at 4 °C. Skin sample for microarray analysis was stored in RNAlater™ (Invitrogen). Samples were kept cool during transport and microarray samples stored at −80 °C until RNA extraction. The CF was calculated as: CF = 100 × Total body weight (g) × fork length^−3^. To obtain comparable growth measurements between the systems, thermal growth coefficients (TGC) were determined (Table 1), calculated as TGC = ((W_1_^0.333^ − W_0_^0.333^)/(Σ °C)) × 1000, where W_0_ and W_1_ are initial and final weight (g) and Σ °C are sum day-degree during the experimental period. The calculated weight development for the populations was used to provide estimates of the mean weight and TGC of the populations as a whole.

**Table 1:** Mean weight, condition factor and thermal growth coefficient ± SD (n = 15 for sampled fish) of Atlantic salmon in open net-pen and semi-closed containment system (S-CCS) 1 and 4 months (1 M/4 M) post seawater transfer.

	Freshwater	One month post transfer		Four months post transfer		Wilcoxon test	Weight	CF
	Smolt	S-CCS	Net-pen	S-CCS	Net-pen	Groups system:time	*p*-value	*p*-value
Sampled (measured)								
Weight (g)	101 ± 4.2	111.8 ± 23.8	113.3 ± 19.6	407.2 ± 83.4	659.9 ± 289.4	Net-pen:1 M - S-CCS:1 M	0.5202	0.9834
CF	1.15 ± 0.01	0.99 ± 0.06	0.98 ± 0.07	1.10 ± 0.07	1.18 ± 0.23	Net-pen:1 M - S-CCS:4 M	<0.0001	0.0002
TGC	n.a.	n.a.	n.a.	2.87 ± 0.67	2.87 ± 1.07	Net-pen:4 M - S-CCS:1 M	<0.0001	0.0212
Production (estimated)						Net-pen:4 M - S-CCS:4 M	0.0619	0.2451
Weight (g)	n.a.	136	132	444	733	Net-pen:1 M - Net-pen:4 M	<0.0001	0.0135
TGC	n.a.	n.a.	n.a.	3.04	2.80	S-CCS:1 M - S-CCS:4 M	<0.0001	0.0005

Comparisons of mean weight and condition factor between each pair were assessed by Wilcoxon.

### Examination of skin tissue sections

Skin samples (n = 6 per time-point, per system) were dehydrated through graded series of ethanol and embedded in paraffin (Histowax, Histolab Products AB). Sections (5 μm) were prepared in the anterior-posterior direction using a Microtom Leica RM 2165 (Leica Microsystems). Parallel sections (n = 2 per fish) were stained with haematoxylin-eosin (HE) (Sigma-Aldrich) and Alcian Blue (Sigma) and Periodic Acid Schiff (Merck) (AB-PAS). Images and measurements were done with Zeiss Axio Observer Z1 equipped with an AxioCam MRc5 camera and AxioVision software (Carl Zeiss Microimaging, GmbH). Overall morphology was examined in HE-stained sections, numbers of mucus cells were counted in AB-PAS images. Total numbers of magenta (neutral) and blue (acidic) mucus cells were counted and the ratios basic:acidic mucus cells calculated. Thickness of epidermis and dermis was measured (n = 10 measurements per sample) using the AxioVision software (Carl Zeiss).

### Microarray

Total RNA was extracted from skin samples using Trizol® reagent (Invitrogen) and purified with Pure Link kits (Invitrogen) including an on-column DNase treatment according to the manufacturer’s protocol. 2100 Bioanalyzer and RNA Nano Chips (Agilent Technologies) was used to verify the integrity of the RNA samples and only samples with RIN values > 7.5 were considered. RNA purity and concentration were measured using a NanoDrop ND-1000 Spectrophotometer (NanoDrop Technologies). Total RNA samples were stored at −80 °C until use. Multiple gene expression profiling in skin was performed using Nofima’s 15 k Atlantic salmon oligonucleotide microarray SIQ-6 (GPL16555) produced by Agilent Technologies; all reagents and equipment were purchased from the same source. Analyses included freshwater smolts (seven individuals) and seawater post-smolts (six individuals per system and time-point), totally 31 arrays were used. Labelling of total RNA (200 ng per reaction) with Cy3 was performed with Low Input Quick Amp Labelling Kit and Gene Expression Hybridization Kit was used for fragmentation. Hybridization was performed for 17 hours in a hybridization oven (Agilent) at 65 °C with a rotation speed of 10 rounds per minute. Arrays were washed for 1 minute with Gene Expression Wash Buffer I at room temperature, and 1 minute with Gene Expression Wash Buffer II at 37 °C and scanned. Gene expression data were processed and analysed with aid of Nofima’s bioinformatics package^21^. In brief, global normalization was performed by equalizing the mean intensities of all microarrays. Next, the individual values for each feature were divided to the mean value of all samples, thus expression ratios (ER) were calculated. Finally, log_2_-ER were normalized with the locally weighted non-linear regression (Lowess). The data are presented as ER to smolts. The dataset was submitted to GEO Omnibus (GSE114028).

### Statistical analyses

Fish weight and CF of the sampled fish (Table 1) were unequal in variance by Welch ANOVA and nonparametric comparisons among each pair were assessed by Wilcoxon (*p* < 0.05). Temperature and salinity measurements between the S-CCS and net-pen systems were compared by a matched pair design using a paired t-test in JMP® v13.1.0. Weight, epidermis, dermis and mucus measurements used in the assessment of histology data were normally distributed. For comparisons of group means we applied two-way ANOVA with post-hoc pairwise multiple comparisons using Tukey honest significant difference (HSD). The gene expression data were compared between smolts (freshwater) and post-smolts (seawater), the systems and time-points. Differentially expressed genes were selected by criteria: *p* < 0.05 and log2-ER > |0.8| (1.74-fold). The study groups are designated by the systems (net-pen and S-CCS) and time in sea (1 M and 4 M; 1 and 4 months, respectively). Correlation was examined between each of the five variables (fish weigh and length, epidermis and dermis thickness and mucus cell numbers), and genes were selected by criteria: Pearson |r| > 0.6, *p* < 0.05, standard deviation of log2-ER > 0.3. STARS categories were counted for genes associated with each of the variables and over-representation of categories was assessed with Fisher’s exact test.

## Results

### Fish and production parameters

Fish weight, cumulated mortality, farming and environmental parameters for S-CCS and the open net-pen systems are illustrated in Supplementary Fig. S1. The temperature increased from 7.5 °C to a maximum of 17.3 °C and 13.1 °C, in the open net-pen and S-CCS respectively. The average measured temperature was 12.9 ± 2.3 °C and 9.5 ± 1.6 °C (mean ± SD) in net-pen and S-CCS, respectively. Daily temperature measurements showed a consistently lower temperature in S-CCS (paired t-test, *p* < 0.0001) with average difference 3.5 °C. The measured salinity was 24.3 ± 1.17‰ and 31.7 ± 1.9‰ (mean ± SD) in net-pen and S-CCS, respectively being at average 7.5‰ higher (paired t-test, *p* < 0.0001) in the S-CCS. The mean body weight estimates (Table 1) from production data was 109 and 113 g when transferred to the open net-pen and S-CCS, respectively. The mean weight of the population 1 and 4 months PST was 132/136 and 733/444 g in the open net-pen and S-CCS, respectively. Recorded weight (Table 1) from individual fish was equal to 113.3 ± 19.6 g (CF = 0.98 ± 0.07) and 111.8 ± 23.8 g (CF = 0.99 ± 0.06) at first sampling and increased to 659.9 ± 289.4 g (CF = 1.18 ± 0.23) and 407.2 ± 83.4 g (CF = 1.10 ± 0.07) after 4 months in net-pen and S-CCS respectively. The S-CCS water current velocity was approximately 0.45–0.90 body length (BL) s-1 1 month PST and decreased to 0.30–0.61 BL s-1 4 months PST. Growth rate of the fish, calculated as TGC, was 2.87 ± 1.07 and 2.87 ± 0.67 (mean ± SD) in net-pen and S-CCS.

### Histological assessments of skin morphology

**Figure 1:**
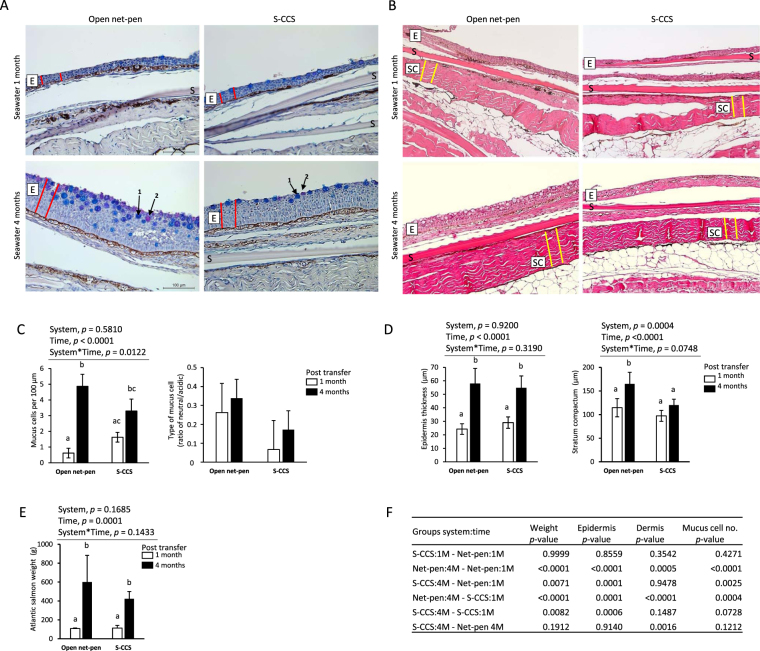
Representative images of Atlantic salmon skin 1 on 4 months post seawater transfer reared in net-pen or semi-closed containment systems (S-CCS). (**A**) Images displaying mucus cells assessed on alcian blue-periodic acid Stiff (AB-PAS) stained sections. AB-PAS stains the mucins in the goblet cells: acidic mucins stain blue (arrow 1) and neutral mucins stain pink-red (arrow 2). Epidermis layer indicated by a boxed E was measured by thickness (μm) as illustrated by red lines. S, scale. (**B**) Differences in the dermis *stratum compactum* (boxed SC) layer thickness (μm) was measured as illustrated by yellow lines. (**C**) Cells stained positive by AB-PAS were counted as goblet cells and presented as mucus cells per 100 µm. Numbers of mucus cells are different with time. The presence of different types of mucus cells is presented as the ratio between neutral and acidic stained cells. (**D**) Quantitative assessments (n = 10 measurements per sample) showing an increase in mean epidermal thickness between time-points but not systems. Thickness in *stratum compactum* of net-pen fish 4 months post seawater transfer was different to other groups. (**E**) Weight of Atlantic salmon used for histology sampling increased between time-points. Bars in all histograms represent the mean ± SD (n = 6 biological samples). Plots were analysed with two-way ANOVA (results in C-E above each graph where one or both variables are significant) and *post hoc* differences tested by Tukey HSD. Bars not sharing common letters are significantly different (*p* < 0.05). (**F**) Pairwise multiple comparisons of weight, epidermis, dermis and mucus measurements showing *p*-values from Tukey HSD.

Corresponding sections of skin were compared between fish reared in open net-pen and S-CCS, 1 and 4 months PST (Fig. 1). The overall tissue morphology and cell integrity, observed in HE stained samples, was in concordance to what is normally observed in skin samples from healthy farmed salmon. No deviations or signs of ulceration or inflammation were observed. To calculate the number of mucus cells we stained the sections with AB/PAS that differentiates between acidic and neutral mucus cells, by staining the mucus blue or magenta, respectively. The number of mucus cells (Fig. 1AC) changed with time in seawater (two-way ANOVA, *p* < 0.0001). However, the pairwise comparison showed an increase in mucus cell numbers between the time-points in open net-pen fish, while there was no difference in S-CCS (Fig. 1CF). No overall difference in mucus cell numbers was observed between fish in open net-pen and S-CCS at the same time-point (Fig. 1F). The ratio of neutral and acidic mucus cells was not different between the systems and sampling points (Fig. 1C). The mean epidermal thickness increased from 24.3 ± 3.9 µm to 58.0 ± 11.2 µm in samples from the open net-pen and from 29.1 ± 4.2 µm to 54.8 ± 8.8 µm in the S-CCS between 1 and 4 months PST (Fig. 1BD). Epidermal thickness was different between time-points in each system (two-way ANOVA *p* < 0.0001), but not between the production systems (two-way ANOVA, *p* = 0.9198). Epidermis of fish in both systems 1 month PST was different to both systems 4 months PST (Fig. 1F). The thickness of the dermis was different between systems (two-way ANOVA *p* = 0.0004) and time-points (two-way ANOVA *p* < 0.0001). The mean dermal thickness increased from 114.6 ± 19.3 µm to 164.8 ± 24.6 µm in samples from the open net-pen and from 97.2 ± 11.6 µm to 120.1 ± 12.5 µm in the S-CCS between 1 and 4 months PST (Fig. 1D). The dermis was thicker in open net-pen fish at 4 months post seawater to the other three groups, which were not different from each other (Fig. 1F).

### Gene transcription profiles

**Figure 2:**
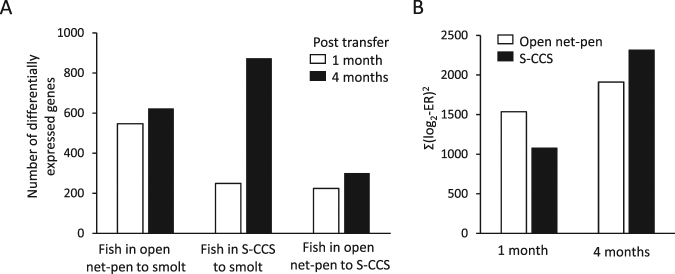
Transcriptome changes in skin of Atlantic salmon post-smolts. (**A**) Numbers of differentially expressed genes comparing smolts in freshwater to post-smolt in open net-pen and S-CCS, 1 and 4 months post seawater transfer. The number of differently expressed genes in skin of Atlantic salmon between the two rearing systems at 1 and 4 months post seawater transfer is shown to the right. (**B**) Magnitude of expression changes in comparison with smolts.

Microarray analyses were designed to compare the skin of Atlantic salmon smolt in freshwater to post-smolt in seawater 1 and 4 months post transfer to either a conventional open net-pen or S-CCS. Figure 2A shows the number of differently expressed genes in post-smolts compared to smolts, which increased in numbers between 1 and 4 months PST for both rearing systems. There was also an increase in the number of differently expressed genes between the two rearing systems between 1 and 4 months PST. At 1 month PST, more genes were differently expressed (Fig. 2A) with a greater magnitude of differential expression (Fig. 2B) in the open net-pen compared to S-CCS. At 4 months PST, the S-CCS produced higher values compared to the open net-pen by both indicators.

**Figure 3:**
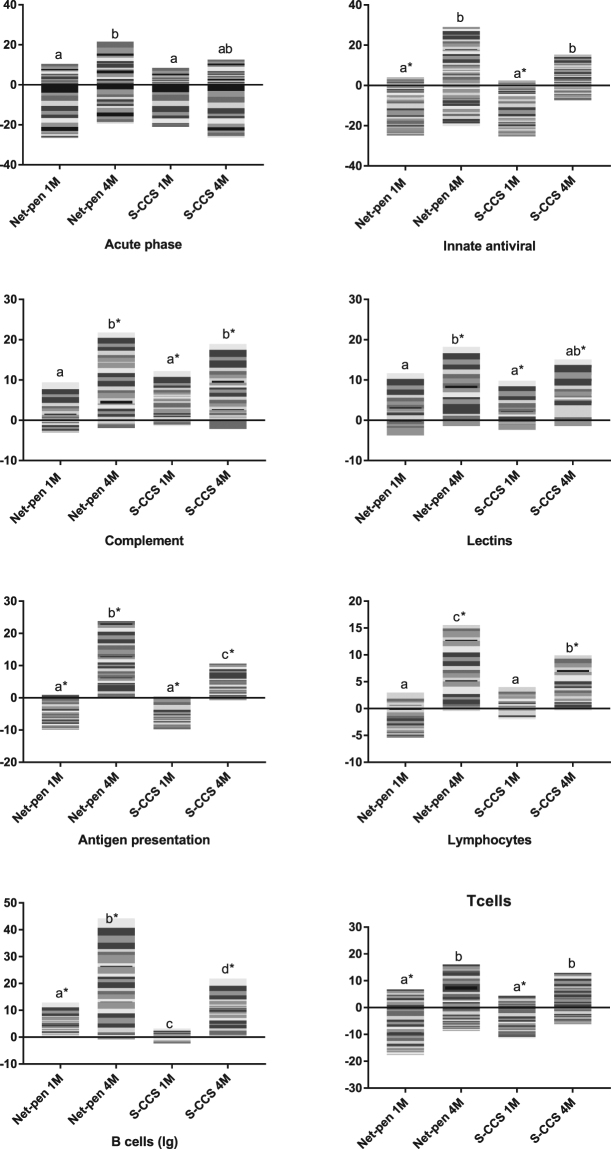
Expression profiles from microarray data in skin of Atlantic salmon reared in either net-pen or semi-closed containment systems (S-CCS) sampled 1 (1 M) and 4 (4 M) months post seawater transfer. Groups of genes involved in immunity. Stacked columns represent log_2_-ER to smolts. Each band corresponds to a gene (microarray feature) presented in a white to black scale, but which is not comparable across functional groups. Comparisons between smolts (freshwater) and post-smolts (seawater), systems and time-points (in seawater) were performed. Bars not sharing common letters are different (*p* < 0.05 and log2-ER > |0.8| (1.74-fold)), difference from smolts in freshwater is indicated with*.

**Figure 4:**
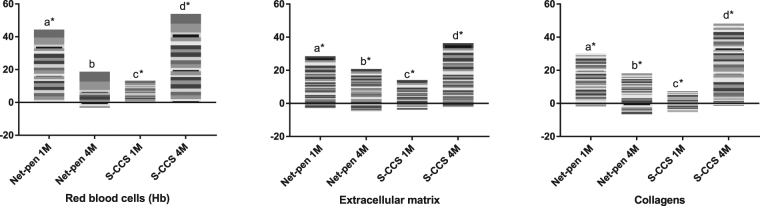
Expression profiles from microarray data in skin of Atlantic salmon reared in either net-pen or semi-closed containment systems (S-CCS) sampled 1 (1 M) and 4 (4 M) months post seawater transfer. Genes encoding markers of erythrocytes, proteins of extracellular matrix and collagens. Stacked columns represent log_2_-ER to smolts. Each band corresponds to a gene (microarray feature) presented in a white to black scale, but which is not comparable across functional groups. Comparisons between smolts (freshwater) and post-smolts (seawater), systems and time-points (in seawater) were performed. Bars not sharing common letters are different (*p* < 0.05 and log2-ER > |0.8| (1.74-fold)), difference from smolts in freshwater is indicated with*.

**Figure 5:**
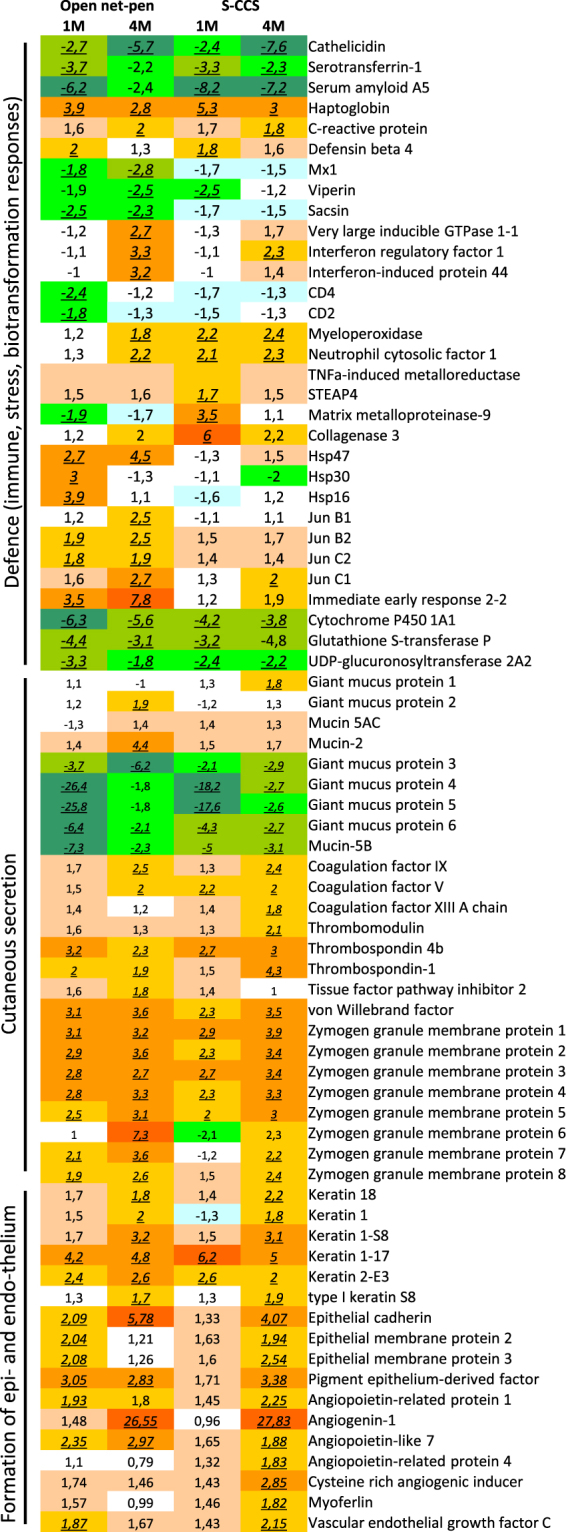
Examples of differently expressed genes involved in defence (immune and stress responses, biotransformation), cutaneous secretion and formation of epithelium and endothelium. Data are folds to freshwater smolts, differently expressed genes (>1.74-fold, *p* < 0.05) are indicated with underlined italics.

Overall, 1816 genes showed differential expression in at least one comparison. Multiple functional groups of genes were different between post-smolts and smolts (Figs 3–5). A large proportion, exactly 345 genes, were related to immune responses, of which 266 (77.1%) were up-regulated in comparison with smolts prior to sea transfer. Genes were grouped by their roles to summarize changes in different parts of the immune system (Fig. 3). At 1 month PST, a decrease was detected in three functionally related groups: innate antiviral responses, antigen presentation (mainly via MHC I) and T cells activity. In contrast, increase was shown by markers of B cells (immunoglobulins) in open net-pen fish, and complement and lectins in S-CCS fish. All groups of immune genes increased expression levels 4 months PST. This trend was more pronounced in the net-pen and differences between the systems emerged in three groups: antigen presentation, lymphocytes (expression was equal at 1 month post transfer) and immunoglobulins. With respect to individual genes, most down-regulated genes were acute phase proteins (e.g. serum amyloids and serotransferrins) known as potent mediators of inflammation in salmon (Fig. 5). Opposite changes were seen in several highly responsive antiviral genes that are commonly co-expressed. A suite of immune effectors with different roles were activated. It is noteworthy to mention different profiles of matrix metalloproteinases *mmp9* and *mmp13*, which at 1 month post transfer were activated only in the S-CCS. Several stress genes with other modes of action had higher expression in control net-pen: chaperones (heat shock proteins) 1 month post sea transfer, *jun* transcription factors and the *immediate early response 2* gene (the most induced gene) 4 months post sea transfer. It is noteworthy that genes with major roles in biotransformation and exposure to chemical stressors i.e. oxidases of xenobiotics and phase II enzymes that increase solubility of lipophilic compounds, were down-regulated.

Complement was consistently up-regulated in salmon post-smolts. Difference to freshwater smolts was significant 1 month post transfer in S-CCS, which increased further in both systems at 4 months post transfer (Fig. 3). Complement was co-regulated with multiple secretory proteins, such as components of the coagulation cascade, which was also stimulated in concert with a suite of *zymogene granule membrane protein* genes that are most likely involved in exocytosis of secretory products (Fig. 5). Down-regulation after seawater transfer was shown by several genes encoding mucosal proteins; moderate increase with respect to smolts was shown by two genes denoted as giant mucosal proteins^22^ or *gmp* and *muc2* (Fig. 5). A hallmark of enhanced activity in skin after seawater transfer was markedly increased abundance of transcripts for erythrocyte markers (mainly *haemoglobin* genes). Salmon from the open net-pen and S-CCS showed different expression profiles. In the former, sharp increase was followed with marked decline but which was still from smolt, while an opposite trend was seen in S-CCS (Fig. 4). Similar profile was observed in several functional groups playing major roles in formation of skin, such as collagens and other protein components of ECM (respectively 36 and 37 transcripts). Steady increase was shown by structural and regulatory proteins specific for epithelium and endothelium with no difference between the systems (Fig. 5).

#### Correlation between morphological variables and gene transcription patterns

**Figure 6:**
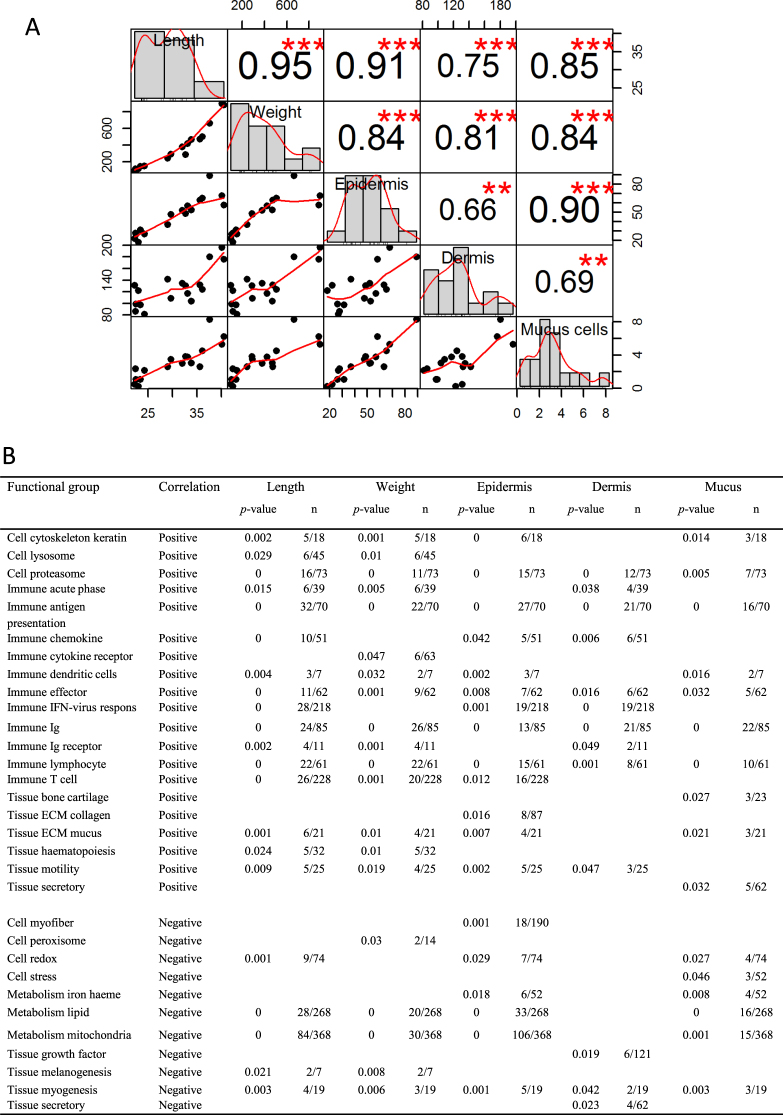
Correlation between physiological variables and gene transcription patterns. (**A**) Chart showing patterns of correlations across fish length, fish weight, skin epidermis thickness, skin dermis thickness and number of skin mucus cells. The distribution of each variable is shown on the diagonal. The bottom part of the diagonal shows scatter plots of the physiological measurements with a fitted line displayed. The top part shows the Pearson correlation coefficients for all pairs of variables including significant correlations marked by red stars with *p*-value: ***<0.001, **<0.01. (**B**) Results for positively followed by negatively correlated genes to the five variables. Fisher-test *p*-values are shown (all results for *p* > 0.05 were removed). Numbers of correlated genes are shown in column “n” together with the total number of genes in the respective category on the array.

We further assessed if any of the measured variables of fish length, weight, epidermis, dermis and mucus cell number could help explain development of skin PST. The analyses provided correlation between all morphological characteristics (Fig. 6A), thus making further plausible explanations difficult. Despite the common trend described between all variables, it is interesting to observe that weight showed inter-correlation with epidermis, dermis and mucus cell numbers (r = 0.81–0.84, *p* < 0.001). Mucus cell numbers correlate strongly to epidermis (r = 0.90, *p* < 0.001) compared to a more moderate correlation to dermis (r = 0.69, *p* < 0.01). Figure 6B shows the comparisons between physiological measured variables on gene transcription levels within functional related gene groups. Utilizing the physiological variables allowed gene groups to be associated separately to different skin structures such as mucus numbers, epidermis or dermis. Overall, 20 and 11 functional groups correlate positively or negatively to the variables. Immune groups correlate in general positively to length and weight, with a more diverse pattern to epidermis, dermis and mucus cell numbers. The tissue ECM collagen group was positively correlated to epidermis (*p* = 0.016, 8/87 (no. of correlated genes to the total no. of genes in the category)). The ECM mucus group correlates positively to epidermis (*p* = 0.007, 4/21) and mucus (*p* = 0.021, 3/21). The tissue secretory group correlate positively to mucus (*p* = 0.032, 5/62), but negatively to dermis (*p* = 0.023, 4/62). In terms of skin development, it can also be of interest that genes involved in metabolism of iron haem (*p* = 0.018, 6/52), lipid (*p* < 0.001, 33/268) and mitochondria (*p* < 0.001, 106/368) correlate negatively to epidermis.

## Discussion

Increased production in closed or semi-closed rearing systems is a strategic focus to reduce the risk of infectious sea lice infestation in the Norwegian Atlantic salmon aquaculture. Assessment of the growth and health performance of salmon in such rearing units is of high importance. An important limitation of the S-CCS investigated here was reduced water temperature during the summer months due to water intake below the sea lice belt. Given equal TGC in both systems, the difference of growth was explained mainly with difference in water temperature from deeper water intake (30 m). This agrees with previously findings reporting temperature and not salinity (28 and 34‰ salinity, at 4 and 8 °C temperature regimes) as a factor for growth rate during the first 2 months in seawater^23^. Still, effect of salinity on growth cannot be entirely excluded at higher temperature and longer time intervals as increased salinity could enhance energy expenditure^24–26^. Raceway systems are designed to control water velocity, which is known to affect growth performance^27,28^. The S-CCS investigated in this study provided constant current in contrast to the variable conditions in the open net-pen. In terms of growth performance, adjusted for temperature, no differences were found between the systems in our study.

Skin biopsies were used to study gene transcriptional changes, and gene group expression levels in relation to time PST and to morphological measured variables (by histology) of the skin. Gene expression changes suggest profound similarity of developmental processes that took place in skin of salmon reared in both systems. The observed gene expression profiles between systems can be categorized as: (i) identical or similar, (ii) similar temporal changes with greater magnitude in open net-pens and (iii) stimulation in both systems with shift in time. Equal expression changes were observed in genes encoding mucus components and proteins that control secretory processes being in line with the increased numbers of mucus producing cells. Microarray data, as also indicated by acidity measurements (AB-PAS staining), suggested modification of the composition of mucus. We previously defined a novel group of genes, which are specific for fish, denoted as giant mucus protein (GMP) that encode high molecular weight multi domain proteins^22^. Here, several GMPs were down-regulated after seawater transfer, while a number of genes for other mucosal proteins were up-regulated indicating a change in mucus composition during transition to seawater. We have also shown that mucus composition in salmon skin is affected by other external parameters, like density and stress (Sveen *et al*., in prep), indicating that properties of mucus may alter in response of external cues.

Co-expression of coagulation cascade and complement is commonly observed in Atlantic salmon skin^29^ and provides a link between the secretory and immune activities. The immune status of salmon was of special importance in this study since recent transcriptome analyses in the head kidney, gill and intestine showed massive down-regulation of immune genes during smoltification, which persisted three weeks after seawater transfer^18^. Here, large-scale expression changes confirmed active immune role of salmon skin. Decreased transcription in comparison with smolts was observed 1 month PST mainly in three functional groups of immune genes, two of which (innate antiviral and T cells immunity) were previously shown to be most affected during smoltification^18^. At 4 months PST, an increase was observed in all groups suggesting that 4 months were sufficient for complete recovery and further stimulation of immunity. The effect was greater in the net-pen, which resulted in difference between the systems by several functional groups including antigen presentation, lymphocytes and B cells. Low temperatures tend to suppress defence in fish and adaptive immunity is especially sensitive^13,30^. This correlates to the increase in skin deterioration and susceptibility to infectious diseases at low seawater temperatures^3^. Given similarity of transcription profiles, fish in the S-CCS most likely required more time to reach the same level of resilience than those farmed in open net-pens, this effect may however be associated with temperature.

Skin thickness equally augmented in pace with the increase of body size, irrespective of the difference in mean temperature between the systems. Results presented here contradict a recently reported inverse relationship between epidermal thickness and temperature^31^. To the best of our knowledge, we are unaware of any additional reports on epidermis thickening in Atlantic salmon and if this is a normal developmental feature during the first time-period at sea. Long term effects of normal production stressors such as crowding and handling may be considered, as epidermal parameters and thickness of fish skin may be restored or even increased after exposure to acute stressors^32,33^. From the transcription analysis, enhanced expression indicates processes that might be prioritized in salmon skin after seawater transfer. Substantial highly co-ordinated changes took place in three functional groups related to the development of skin: erythrocyte markers that most likely indicated circulatory activity of skin, collagens and other protein components of ECM. Unlike genes involved in secretion and immunity, expression profiles were different between the systems. In S-CCS, expression profiles mirrored increase of epidermis thickness. Expression of two MMPs or collagenases (*mmp9* and *mmp13*), were in converse relationships to the collagen and ECM group. MMPs are actively involved in inflammatory and stress responses in Atlantic salmon^34^. Their activity may be central in collagen reorganization during wound repair in fish^35^ and ECM degradation during processes of skin remodelling^36,37^. The dermis layer of fish is primarily connective tissue infiltrated by fibroblasts, in which collagen fibres are an abundant component^38^. The transcription levels indicate a lower level of collagens in the skin of the S-CCS fish in line with the thinner dermis in S-CCS compared to open net-pen fish (4 months PST). Both swimming and temperature may affect ECM production, including collagens, in the vertebrae of Atlantic salmon^39,40^. Skin is likely similar or even a more adaptive tissue compared to bone. Thus, transcriptional differences of collagens and other genes of the ECM group between the two rearing systems could be associated to differences in system design. An alternative explanation to the contradictive expression profiles and microscopic observations is that there may be a relatively low speed of developmental processes and consequently time-lag between enhancement of genes expression and the visible changes.

In this study, microarray technology was used to examine the expression of thousands of genes simultaneously and histology used to measure physical dimensions and mucus characteristics of skin tissue. Skin of Atlantic salmon is a complex tissue and as most tissues composed of a mixture of different cell types. Although the site of the skin biopsy has been standardized there will be individual variances with a difference in tissue structure and cell types. This could provide inconsistent and imprecise measurements used to evaluate the tissues properties and influence the gene expression profiles used to describe functions, pathways and regulatory mechanisms at a given point in time. The microarray hybridization approach only allows a limited dynamic range of detection, and only sequences that are included on the array are detected making expressed non-complementary sequences lost in the analysis. In addition, the probes may not uniquely detect specific genes as related sequences may bind to one probe or a specific sequence to multiple probes. The individual variability and the technological limitations may render the results inaccurate and effect the clinical relevance of this study.

Therefore, a multidisciplinary approach was used to integrate microarray results with histological data sets and also production data like fish size. The combined dataset revealed groups of genes that may be involved or correlated to the morphological data. Some comparisons correlate well with what can be expected, such as mucus cell numbers were positively correlated to genes involved in ECM and mucus production and secretion. It is further indicated that gene groups of haem and lipid metabolism and mitochondria in the skin are negatively correlated to growth (length, and weight). Correlation analyses reveal these changes to be associated to the morphological restructuring of the epidermis and not the dermis part of the skin. Indicating that epidermis cells have at least two interacting gene networks between metabolism and the inversely expressed immune genes. Similar to the reported interplay in the intestinal epithelium of immune dysfunctional mice that increase immune functions at the expense of metabolic activity^41^.

To conclude, the differences in size between open net-pen and S-CCS Atlantic salmon are most likely linked to differences in water temperature between systems. Results suggest that epidermal and dermal thickness and mucus cell numbers increase with a gradual recovery of immune activity in skin PST. These immunosuppressive effects on skin PST may be of great importance to the integument defence mechanisms, since ulceration is assigned as the main probable cause of death in cases where infectious agents are involved^2^. In future studies, we propose to investigate when expression of immune genes exceeds the levels observed in smolts. Longer period of observations with larger number of time-points should be prioritized. Overall, our conceptual interpretation is that the period PST should be considered as a *barrier recovery phase* where the fish builds resilience and robustness for further growth. Temporary immune suppression is a strong argument for using rearing facilities that reduce encounter with fish pathogens during the first months in the sea.

## Electronic supplementary material

Supplementary Figure S1
