# Supplementary material for: Atlantic salmon skin barrier functions gradually enhance after seawater transfer
Source: Sci Rep. 2018 Jun 22;8:9510. doi: 10.1038/s41598-018-27818-y (PMC6015023; doi:10.1038/s41598-018-27818-y)
Supplement: Supplementary file 1 — Supplementary Figure S1 [file 41598_2018_27818_MOESM1_ESM.docx]

**Supplementary information**

Atlantic salmon skin barrier functions gradually enhance after seawater transfer

Christian Karlsen^1*^, Elisabeth Ytteborg^1^, Gerrit Timmerhaus^1^, Vibeke Høst^1^, Sigurd Handeland^2^, Sven Martin Jørgensen^1^, Aleksei Krasnov^1^

^1^Nofima, Osloveien 1, 1430 Aas, Norway

^2^UNI Research, Nygårdsgaten 112, 5008 Bergen, Norway

^*^Corresponding author, [christian.karlsen@nofima.no](mailto:christian.karlsen@nofima.no)


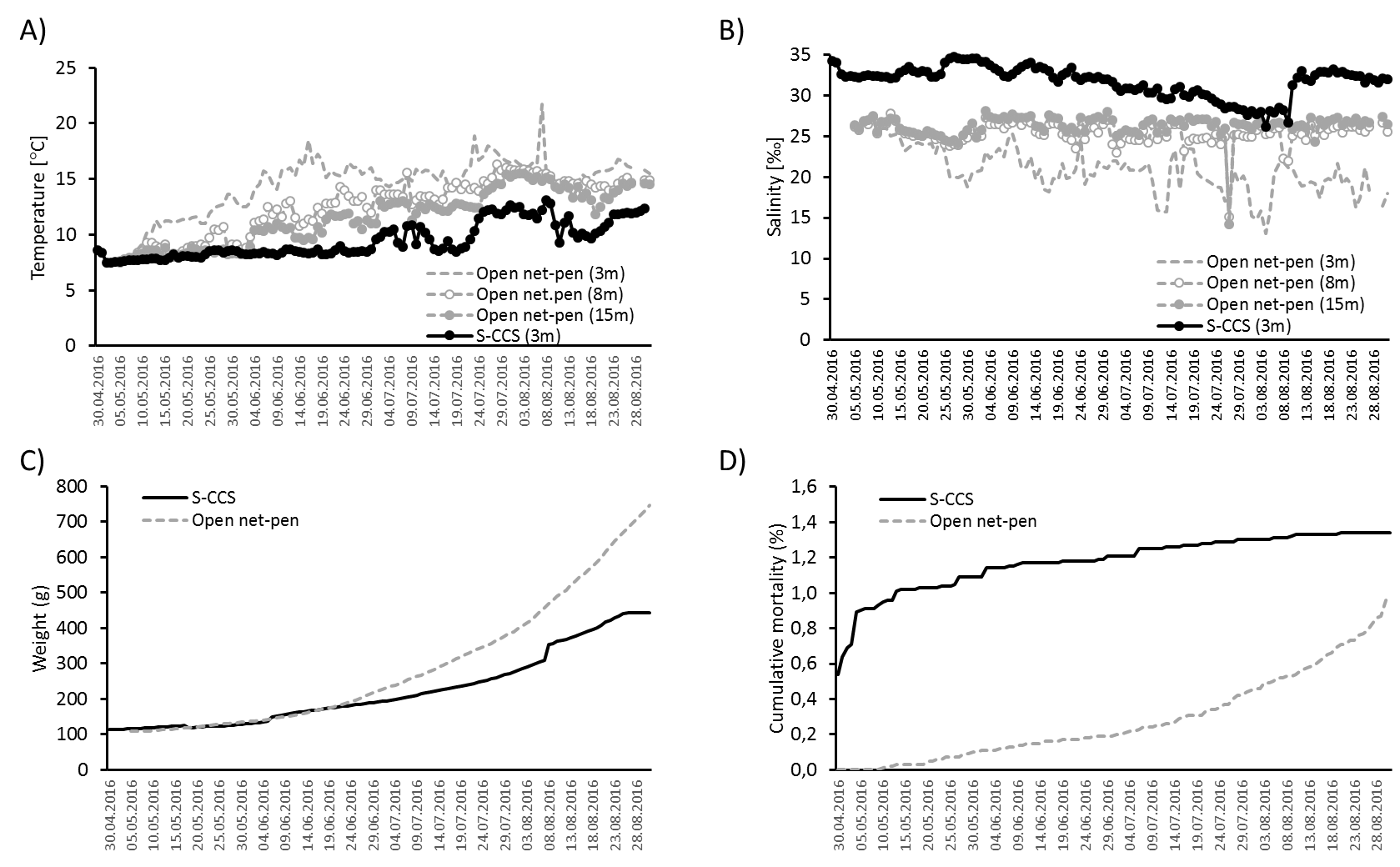


**Supplementary Figure S1.** Fish, farming and environmental parameters for the S-CCS and the open net-pen. Seawater temperature **A**) and salinity **B**) measured daily at different depths. **C**) The Atlantic salmon average weight as measured daily. **D**) Cumulative mortality during the experimental period.
